# E-Scooter-Unfälle und deren Folgen: Erste prospektive Analyse der Verletzungsrate und Verletzungsmuster im urbanen Raum einer deutschen Millionenstadt

**DOI:** 10.1007/s00113-020-00910-7

**Published:** 2020-10-27

**Authors:** Olivia Mair, Markus Wurm, Michael Müller, Frederik Greve, Sebastian Pesch, Dominik Pförringer, Peter Biberthaler, Chlodwig Kirchhoff, Michael Zyskowski

**Affiliations:** Klinik und Poliklinik für Unfallchirurgie, Klinikum rechts der Isar, Technische Universität München, Ismaninger Str. 22, 81675 München, Deutschland

**Keywords:** E‑Scooter, Traumafolgen, Ballungsraum, Alkoholisierung, Notaufnahme, E‑scooter, Trauma mechanism, Head injury, Alcohol, Emergency room

## Abstract

**Hintergrund:**

Seit ihrer Zulassung in Deutschland im Juli 2019 erfreuen sich E‑Scooter zunehmender Popularität. Diese steigende Beliebtheit und die einfache Zugänglichkeit der E‑Scooter für jedermann haben jedoch auch zu teils schweren Unfällen geführt.

**Ziel der Arbeit:**

Das Ziel dieser Studie war es, die Art und Schwere der Verletzungen, die in direktem Zusammenhang mit der Nutzung von E‑Scootern in einer deutschen Millionenstadt stehen, zu analysieren und hieraus Schlüsse für zukünftige Sicherheitskonzepte und Verhaltensregeln zu ziehen.

**Methodik:**

Alle Patienten, die sich aufgrund eines Unfalls mit einem E‑Scooter in der interdisziplinären Notaufnahme des Klinikums rechts der Isar, Universitätsklinikum der Technischen Universität München, zwischen dem 01.07.2019 und dem 01.04.2020 vorstellten, wurden erfasst und sowohl demografische Daten als auch Informationen zu Traumamechanismus und den entstandenen Verletzungen dokumentiert.

**Ergebnisse:**

Im oben genannten Zeitraum wurden prospektiv 60 Patienten erfasst, wovon 34 (56,7 %) Männer waren. Durchschnittlich waren die Patienten 34,7 Jahre (18 bis 73 Jahre) alt. Unter Alkoholeinfluss fuhren 22 Patienten (36,7 %); ein Helm wurde lediglich von einer Person getragen. Verletzungen des Kopfes waren mit Abstand am häufigsten, gefolgt von Verletzungen der oberen und der unteren Extremität (Radiusköpfchenfraktur *n* = 5, Riss-Quetsch-Wunden an Fuß/Sprunggelenk *n* = 8). 2 Patienten (3,3 %) waren schwer verletzt (ISS ≥16)

**Diskussion:**

Mit zunehmender Beliebtheit der E‑Scooter steigt auch die Anzahl an Verletzungen. Am häufigsten ist die Kopfregion betroffen, weshalb zukünftig eine Helmpflicht sinnvoll erscheint. Zudem sollten eine breitere Informationskampagne und strengere polizeiliche Kontrollen im Hinblick auf die Vielzahl an alkoholisierten Unfallopfern erfolgen.

## Abstract (en)

**Background:**

Since their approval in Germany in June 2019 e‑scooters have become increasingly more popular; however, with growing popularity and easy access for everybody the number of accidents has increased, sometimes even resulting in severe injuries.

**Objective:**

The purpose of this study was to collect demographic data and analyze injury patterns of accidents due to e‑scooter use in a large German city and to give suggestions on future safety concepts and rules of conduct.

**Methods:**

All patients who presented at the interdisciplinary emergency room of the Klinikum rechts der Isar, University hospital of the Technical University in Munich between 1 July 2019 and 1 April 2020 after suffering an e‑scooter accident were included in this study. Demographic data as well as information with respect to the trauma mechanism were prospectively collected.

**Results:**

During the study period 60 patients were identified of which 34 (56.7%) were male with an average age of 34.7 years (range 18–73 years) at the time of the accident. Of the patients 22 rode the e‑scooter under the influence of alcohol and only 1 wore a helmet. The head was the most injured body region, followed by the upper and lower extremities and 2 (3.3%) patients were severely injured with an injury severity score (ISS) ≥16.

**Conclusion:**

Due to the growing popularity of e‑scooter use more injuries have been registered. The head was the most affected body region, therefore emphasis should be put on wearing a helmet. Furthermore, strict rules and more police controls should take place to identify and reduce the number of accident victims under the influence of alcohol.

## Einleitung

Die fortschreitende Urbanisierung, begleitet durch eine kontinuierliche Steigerung des innerstädtischen Verkehrsaufkommens, schafft ein zunehmendes Platz- und Umweltproblem in modernen Großstädten [1, 2]. Gleichzeitig wird der Wunsch der Gesellschaft nach nachhaltigen, klimaeffizienten Transportmöglichkeiten ohne Einschränkung der individuellen Mobilität größer [3]. Mit Zunahme der „sharing economy“ hat sich die vermeintlich umweltfreundliche Sparte der Elektromobilität in Form von E‑Scootern basierend auf dem Ride-Sharing-Prinzip etabliert [4].

E‑Scooter wurden erstmals in Santa Monica, Kalifornien, im Juni 2017 im kommerziellen Setting der Öffentlichkeit zur Verfügung gestellt. Auf Deutschlands Straßen sind diese seit dem Inkrafttreten der „Elektrokleinstfahrzeuge-Verordnung“ am 15.06.2019 zugelassen und prägen immer mehr das Stadtbild der Ballungszentren [5]. Momentan werden in München von 6 verschiedenen Anbietern mehrere Tausend E‑Scooter zum Verleih angeboten [6].

Die vor Einführung der E‑Scooter in Deutschland bekannten internationalen Unfalldaten ließen erahnen, dass mit einem Anstieg an Unfällen mit teils schweren Verletzungen zu rechnen sein wird [7–9]. So berichteten Namiri et al. in den USA zwischen 2014 und 2018 von einem prozentuellen Anstieg von 222 % der Verletzungen durch E‑Scooter-Unfälle sowie einen Anstieg der Hospitalisierungen um 365 % [7]. Ishmael et al. untersuchten wiederum die unfallchirurgischen Verletzungen, die nach E‑Scooter-Unfällen in einem „trauma center“ in Kalifornien innerhalb von 2 Jahren operativ versorgt werden mussten. Sie beobachteten bei den insgesamt 75 operativ zu versorgenden Frakturen eine Ähnlichkeit zu Verletzungsmustern, die sonst nur im Rahmen von Hochrasanztraumen auftreten [8]. Eine retrospektive Analyse von Kobayashi et al. zeigte in einem Zeitraum von 14 Monaten bei 103 Patienten einen mittleren Injury Severity Score (ISS) von 5,9 [10, 11]. Trivedi et al. fanden in der bisher größten E‑Scooter-Studie mit 249 Fällen, dass nur 6 % der Patienten bei Fahren des E‑Scooters und entsprechend zum Zeitpunkt des Unfalls einen Helm trugen. 5 % der Unfälle ereigneten sich unter Alkoholeinfluss [9]. Die Rate an alkoholisierten Unfallopfern war in der bisher ersten deutschsprachigen Fallserie mit 17 % bei 24 eingeschlossenen Patienten sogar noch höher [12].

### Technischer Hintergrund

**Figure 1:**
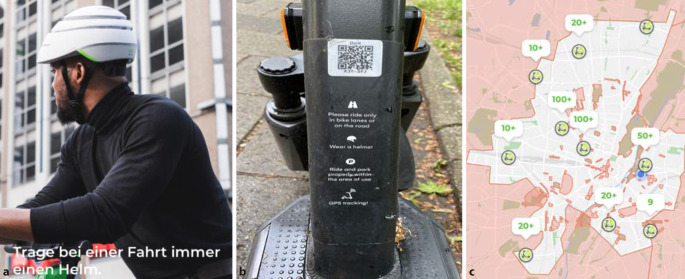

Für die Miete eines E‑Scooters muss der Benutzer eine App auf sein Smartphone laden, auf der alle verfügbaren E‑Scooter des jeweiligen Anbieters auf einer Karte per GPS angezeigt werden [13]. Via Scan eines QR-Codes kann jeder abgestellte E‑Scooter nach Bezahlung einer Startgebühr von ca. 1 € aktiviert und dann für eine minütliche Pauschale (je nach Anbieter ca. 0,15–0,25 €) benutzt werden. Die E‑Scooter können nach der Benutzung überall innerhalb der designierten Sharing-Zone abgestellt werden und stehen sofort dem nächsten User zur Verfügung (Abb. 1c). Die Nutzung erfolgt v. a. auf kurzen Strecken und wird als Transportmittel für die bekannte „letzte Meile“ genutzt [10].

Das Bundesministerium für Verkehr und digitale Infrastruktur (BMVI) schreibt eine Höchstgeschwindigkeit von 20 km/h vor, es wird kein Führerschein benötigt, jedoch ist ein Mindestalter von 14 Jahren ist für die Benutzung obligat [5]. Wenn möglich sollen E‑Scooter auf Radwegen benutzt werden, eine Helmpflicht besteht nicht, sie wird lediglich vom BMVI und den Anbietern empfohlen (Abb. 1a,b; [5]).

Wie beim Führen von Kraftfahrzeugen besteht ein Alkoholgrenzwert von 0,5 ‰ [5]. Verstöße gegen diesen Grenzwert werden auch bei Benutzern von E‑Scootern mit Bußgeldern und einem Führerscheinentzug geahndet [5]. Diese Gesetzeslage führte beispielsweise während des Münchner Oktoberfests 2019 zu verschärften Regelungen mit ausgedehnten Sperrzonen und intensiveren Kontrollen. So wurden durch die Münchner Polizei aufgrund von alkoholisierten Fahrten mit E‑Scootern während diesen 2 Wochen nach eigenen Angaben 254 Führerscheine eingezogen; dies entspricht einer deutlichen Zunahme im Vergleich zum Vorjahr [14].

Ziel der vorliegenden Studie war es, neben der Erhebung und Analyse der demografischen Daten der mit einem E‑Scooter Verunfallten die Traumafolgen und Verletzungsmuster nach E‑Scooter-Unfällen zu analysieren, mögliche Sicherheitsempfehlungen zu formulieren und das medizinische Personal in deutschen zentralen Notaufnahmen auf zu erwartende Verletzungen zu sensibilisieren.

## Studiendesign und Methodik

Im Zeitraum zwischen 01.07.2019 und 01.04.2020 wurden prospektiv alle nach einem E‑Scooter-Unfall in der interdisziplinären Notaufnahme des Klinikums rechts der Isar, Universitätsklinik der Technischen Universität München, vorstellig gewordenen Patienten in diese Studie eingeschlossen. Die demografischen Daten, wie Alter, Geschlecht und der Wohnsitz der Verunfallten, wurden erfasst und analysiert. Zusätzlich erfolgte die Erfassung von Informationen zum Unfallgeschehen wie Tageszeit, Modalität der Vorstellung in der Klinik (Selbsteinweisung/mittels Rettungsdienst/mit Notarztbegleitung/Schockraumeinweisung) und die Notwendigkeit einer stationären Aufnahme. Falls ein stationärer Aufenthalt nötig war, wurden die Länge des Krankenhausaufenthalts und eine mögliche intensivmedizinische Behandlung erfasst. In Bezug auf den Unfallmechanismus wurde zudem erhoben, ob Fremdverschulden der Unfallauslöser war, ob ein Alkoholeinfluss vorlag, ob ein Helm getragen wurde, und ob die Fahrt mit dem E‑Scooter auf dem Arbeitsweg passierte. Die jeweiligen Verletzungen wurden vollständig erhoben, nach Köperregionen katalogisiert und zur differenzierten Betrachtung nach Schwere der Verletzung und Indikation zur operativen Versorgung unterteilt. Zudem wurde die in der Notaufnahme durchgeführte Behandlung erfasst und in Subgruppen geordnet.

Für die statistische Auswertung der Ergebnisse wurde das Programm IBM® SPSS® Version 6 (New York, USA) verwendet.

## Ergebnisse

**Figure 2:**
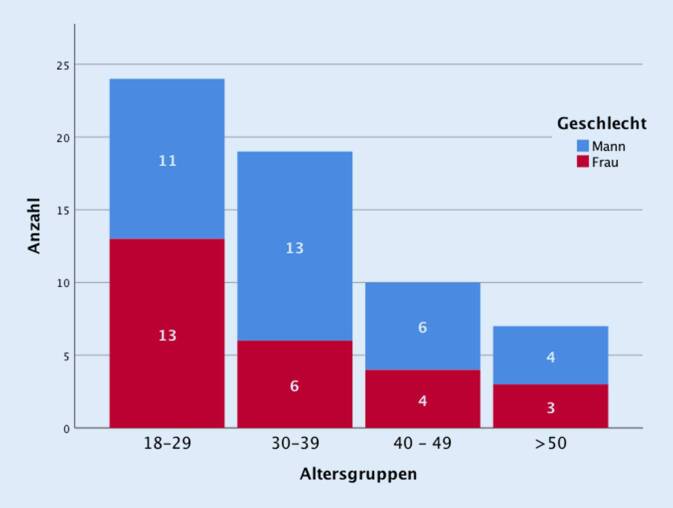

In diese prospektive Studie wurden im Studienzeitraum vom 01.07.2019 bis 01.04.2020 60 Patienten eingeschlossen. Davon waren 34 männlich (56,7 %) und 26 weiblich (43,3 %) (Abb. 2). Zum Zeitpunkt des Unfalls waren die Patienten im Mittel 34,7 Jahren alt (Altersspanne 18 bis 73 Jahre).

40 Patienten (66,7 %) waren in München wohnhaft. Insgesamt verunfallten nur in München wohnhafte Patienten (*n* = 6, 10 %) auf dem direkten Arbeitsweg.

Bezüglich der Verteilung der Unfälle nach Tageszeit zeigten sich keine signifikanten Unterschiede. In 25 % der Fälle (*n* = 15) verunglückten die Patienten in der ersten Tageshälfte (6–14 Uhr), 23 Patienten (38,3 %) in der zweiten Tageshälfte (14–22 Uhr) und 22 Patienten (36,7 %) nachts (22–6 Uhr). 22 Unfälle ereigneten sich unter Alkoholeinfluss (36,7 %), von denen 16 (72,7 %) nachts registriert wurden.

38 Patienten (63,3 %) wurden selbstständig in der Notaufnahme vorstellig, 19 (31,7 %) wurden durch den Rettungsdienst eingewiesen, und 3 Patienten (5,0 %) kamen mit Notarztbegleitung. Insgesamt wurden 4 Patienten (6,7 %) über den Schockraum in die Klinik eingewiesen bzw. aufgenommen. Von den 4 Schockraumeinweisungen erfolgten 3 in Notarztbegleitung; ein Patient wurde aufgrund der räumlichen Nähe des Unfallortes zur Klinik durch den Rettungsdienst ohne Notarzt im Sinne einer „Load-and-go“-Zuverlegung eingeliefert.

Ein stationärer Aufenthalt direkt nach dem Unfallereignis war insgesamt bei 10 Patienten (16,7 %) notwendig, wobei kein Patient auf der Intensivstation betreut werden musste. Im Durchschnitt waren die Verunfallten 6 Tage in stationärer Behandlung (1 bis 26 Tage, Median: 3,5). Nur 5 Unfälle (8,3 %) waren eindeutig auf Fremdverschulden zurückzuführen. Bei 9 Patienten (15 %) konnte aufgrund einer retrograden Amnesie der genaue Unfallmechanismus nicht mehr rekonstruiert werden.

In 98,3 % (*n* = 59) der Fälle waren die E‑Scooter angemietet, wobei kein Patient zum Unfallzeitpunkt einen Helm trug. Zum Zeitpunkt des Unfalls trug nur ein Patient einen Helm, der als Einziger auch Eigentümer des E‑Scooters war (Tab. 1).

**Table 1:** 

Patientencharakteristika des Studienkollektivs (*n* = 60)		
Alter	MW = 34,7 Jahre, RW: 18–73	
	18–29 Jahre	24 (40,0 %)
	30–39 Jahre	19 (31,7 %)
	40–49 Jahre	10 (16,7 %)
	≥50 Jahre	7 (11,7 %)
Geschlecht	Männer	34 (56,7 %)
	Frauen	26 (43,3 %)
Einheimisch	–	40 (66,7 %)
Touristen	–	20 (33,3 %)
Alkoholisierung	–	22 (36,7 %)
Unfallzeitpunkt	6–14 Uhr	15 (25,0 %)
	14–22 Uhr	23 (38,3 %)
	22–6 Uhr	22 (36,7 %), davon unter C2: 11 (78,5 %)
Einlieferungsmodalität	Selbstständig	38 (63,3 %)
	Rettungsdienst	19 (31,7 %)
	Notarztbegleitung	3 (5,0 %)
Behelmung	–	1 (1,7 %)
Stationärer Aufenthalt	–	10 (16,7 %)
Dauer des stat. Aufenthalts	MW = 6,0 Tage; RW: 1–26 Tage	
Schweregrad der Verletzung (anhand des ISS [11])	MW = 3,82; RW: 1–24	
	Leicht: ISS 1–8	54 (90 %)
	Moderat: ISS 9–15	4 (6,7 %)
	Schwer: ISS ≥16	2 (3,3 %)

*MW* Mittelwert, *RW* Reichweite, *ISS* Injury Severity Score

**Figure 3:**
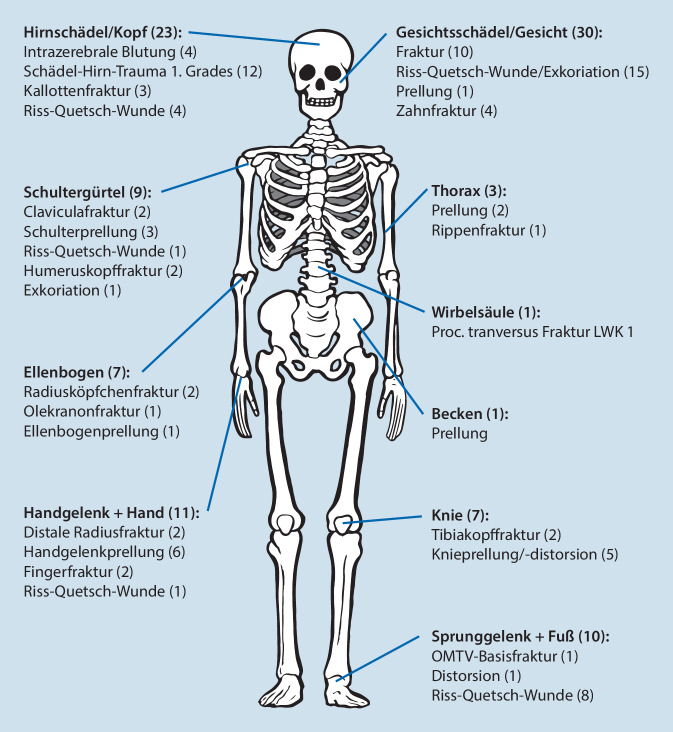

Bei den 60 in diese Studie eingeschlossenen Patienten wurden 102 Verletzungen registriert (Abb. 3). Die meisten Verletzungen, 53 aus 102 (51,9 %), wurden im Kopf- bzw. im Gesichtsbereich detektiert. Außerdem ergaben sich 27 Verletzungen der oberen (26,4 %), 17 an den unteren Extremitäten (16,7 %) und 5 Traumafolgen am Körperstamm (4,9 %). Im Durchschnitt erlitt demnach jeder Verunfallte 1,7 Verletzungen.

Die genauere Betrachtung der entstandenen Verletzungen zeigt, dass 26 Patienten (43,3 %) insgesamt 33 Frakturen erlitten, wobei 15 Frakturen im Bereich der oberen Extremität, 3 an der unteren Extremitäten und eine im Bereich der Wirbelsäule sowie eine im Bereich des Thorax und 13 im Bereich des Kopfes lokalisiert waren, Zahn- bzw. Zahnwurzelverletzungen ausgenommen.

Oberflächliche Hautlazerationen und Riss-Quetsch-Wunden waren bei 26 Patienten (43,3 %) vorhanden, davon erlitten 19 Patienten Läsionen im Bereich des Gesichtes bzw. Kopfes, 8 an den unteren Extremitäten und 2 an den oberen Extremitäten; mehrfache Verletzungen sind hierbei separat aufgeführt.

Zusätzlich wurden bei 4 Patienten (6,7 %) intrazerebrale Blutungen festgestellt, was für die betroffenen Patienten eine engmaschige Monitorüberwachung über 48 h zur Folge hatte. 12 Patienten (20 %) erlitten ein Schädel-Hirn-Trauma 1. Grades (Abb. 3).

Im Rahmen der ambulanten Versorgung in der Notaufnahme erhielten 34 (56,7 %) Patienten eine Röntgenuntersuchung mindestens einer Extremität, wobei in 7 Fällen (11,7 %) eine additive Computertomographie (CT) der betroffenen Extremitäten durchgeführt wurde. Eine Sonographie des Abdomens im Sinne eines FAST-Scans wurde bei 11 Patienten (18,3 %) durchgeführt, eine CT des Gesichtsschädels war bei 18 Patienten (30,0 %) indiziert, und eine CT der HWS und des Hirnschädels war bei 19 Patienten (31,7 %) nötig.

Bei 23 Patienten (38,3 %) war eine Hautnaht nötig, und 18 (30,0 %) Patienten mussten mittels eines Schienenverbands (inkl. Orthesen) versorgt werden.

Insgesamt wurde bei 15 Patienten (25,0 %) aufgrund der erlittenen Verletzungen die Indikation zur operativen Versorgung gestellt. Davon wurden 8 Patienten (13,3 %) im Verlauf insgesamt 10-mal operativ versorgt (Tab. 2); die restlichen nichtortsansässigen Patienten wünschten eine heimatnahe Versorgung.

**Table 2:** 

Verletzungen mit Indikation zur operativen Versorgung	
Angeführt sind jeweils die Art der Verletzung und die entsprechend durchgeführte(n) Operation(en)	
Verletzung	Durchgeführte Operation
Distale Radiusfraktur 2R3-C2 + Distale Radiusfraktur 2R3-C3	Volare Plattenosteosynthese
Laterale Claviculafraktur, Typ Jäger und Breitner IIa	Arthroskopie, CC-Fixation mittels „Tight- rope“-System, Plattenosteosynthese
Tibiakopffraktur Typ Schatzker VI (Abb. 4, 5)	1. Anlage eines Fixateur externe 2. Plattenosteosynthese via dorsomedialem Zugang 3. Plattenosteosynthese via anterolateralem Zugang
Frontobasisfraktur + Orbitadachfraktur + Nasenbeinfraktur	1. Intradurale Frontobasisdeckung mit gestieltem Galea-Periost-Lappen 2. Patch-Einlage in Orbitadach + Reposition Nasenbeinfraktur
Mandibulafraktur	IMF-Schrauben Unterkiefer + Reposition und Osteosynthese der Unterkieferfraktur beidseits
Jochbeinfraktur mit Orbitabeteiligung	Reposition und Osteosynthese Jochbeinfraktur
Nasenbeinfraktur	Nasenbeinreposition und Tamponade

**Figure 4:**
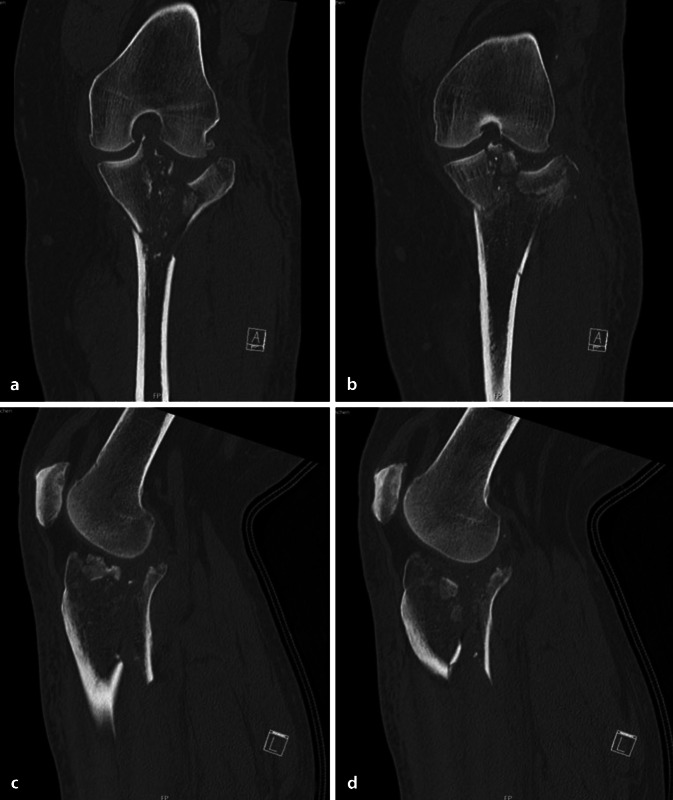

**Figure 5:**
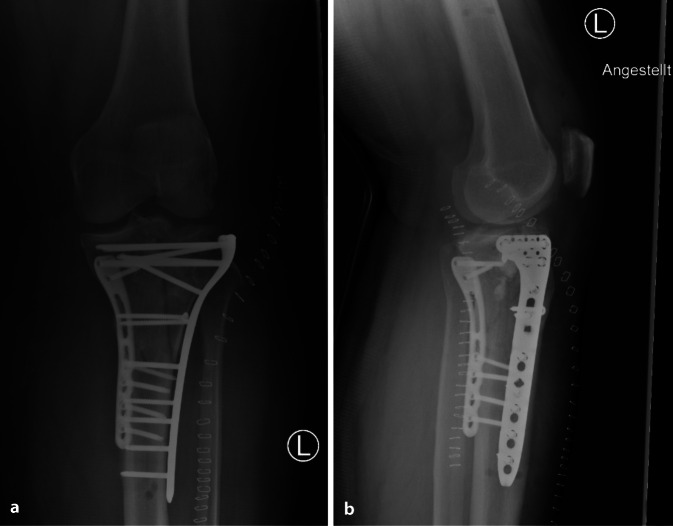

Der durchschnittliche ISS der Verletzungen betrug 3,82 (1–24), wovon 2 Patienten (3,3 %) einen ISS >15 aufwiesen.

**Figure 6:**
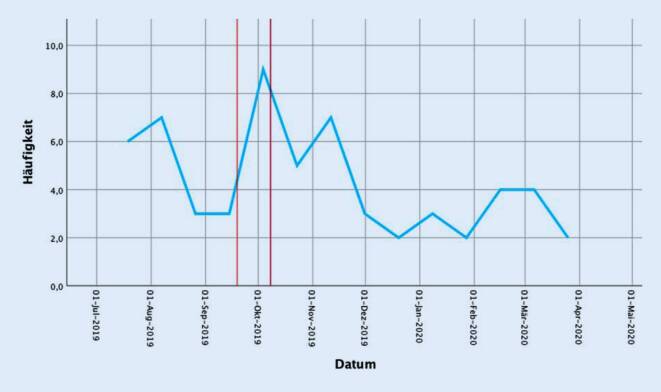

Betrachtet man den Zeitraum des Münchner Oktoberfests gesondert, so fällt auf, dass innerhalb dieser 2 Wochen 9 Patienten mit einem mittleren Alter von 33,1 Jahren (21 bis 46 Jahre) in unserer Notaufnahme nach E‑Scooter-Unfällen vorstellig wurden (Abb. 6). 55,5 % der Patienten (*n* = 5) waren männlich, und der mittlere ISS betrug 6,7 (1–24), dieser Wert ist gegenüber Nicht-Oktoberfest-Zeiten signifikant erhöht. Drei der während des Oktoberfestes eingelieferten Patienten (33 %) mussten stationär versorgt werden, zwei waren schwer verletzt (ISS von 16 bzw. 24). Diese 2 Patienten wurden im Rahmen der stationären Aufnahme über die ersten 24 h einer Monitorüberwachung zugeführt und konnten im weiteren Verlauf des stationären Aufenthaltes auf die Normalstation verlegt werden. Keiner dieser Patienten trug zum Unfallzeitpunkt einen Helm. Die Analyse der während des Oktoberfestes mit einem E‑Scooter Verunfallten ergibt, dass in diesem Zeitraum signifikant mehr Patienten alkoholisiert einen E‑Scooter benutzt als während des restlichen Jahres (66 %, *n* = 6, *p* < 0,05).

## Diskussion

Seit rund einem Jahr, also seit Juni 2019, sind E‑Scooter in Deutschland im Straßenverkehr zugelassen und erfreuen sich seither zunehmender Beliebtheit [5]. Inzwischen bieten immer mehr Unternehmen E‑Scooter auf Basis eines Ride-Share Prinzips an, wodurch diese mittlerweile das Stadtbild vieler deutscher Großstädte prägen. Durch ihre einfache Handhabung, den unbürokratischen Ausleihmodus via App und ihre breite Verfügbarkeit erfreuen sich E‑Scooter großer Beliebtheit, auch wenn sich der Gebrauch in der akuten Coronaphase deutlich reduziert hat [15].

Unsere Studie mit 10-monatiger Laufzeit wurde in der interdisziplinären Notaufnahme des Klinikums rechts der Isar der Technischen Universität München durchgeführt und stellt mit 60 Patienten und insgesamt 102 Verletzungen die bis dato größte Analyse im deutschsprachigen Raum bezüglich Verletzungsmuster, Verletzungsschwere und Behandlungsbedarf von Patienten nach E‑Scooter-Unfällen dar.

Ähnlich wie in bislang publizierten internationalen Studien war der Großteil der E‑Scooter Fahrer jünger, mit einem Alter unter 40 Jahren (71,7 %), sowie überwiegend männlich (56,7 %) [9, 10, 16].

Besonders auffällig erscheint in der Analyse der vorliegenden Daten, dass in unserer Kohorte nur ein Patient (1,7 %) beim Unfall einen Helm trug und dieser als Einziger auch der Besitzer des E‑Scooters war. Diese Beobachtung deckt sich mit Daten aus amerikanischen Studien, die analog zeigten, dass nur 2–5 % der E‑Scooter-Nutzer zum Unfallzeitpunkt einen Helm trugen [9, 10, 17]. In Korrelation dieses Faktums zu der Tatsache, dass mehr als die Hälfte der registrierten Verletzungen (52,0 %, *n* = 53) am Kopf lokalisiert waren, erscheinen die Einführung einer Helmpflicht bzw. regelmäßige und intensive Aufklärungskampagnen zur Helmnutzung bei der Benutzung von E‑Scootern unumgänglich.

Mehrere Anbieter von E‑Scootern empfehlen das Tragen eines Helms; bei einigen erscheint ein Hinweis auch schon beim Ausleihprozess in der App (Abb. 1a,b), die Möglichkeit, einen Helm mit den E‑Scooter zusammen auszuleihen, besteht bei den gängigen Anbietern jedoch nicht.

Betrachtet man Studien aus den USA, wo E‑Scooter schon seit mehreren Jahren zugelassen sind, und zieht die offiziellen Daten der Anbieter hinzu, zeigt sich, dass in den kommenden Jahren mit einem weiteren Anstieg der Verletzungszahlen zu gerechnet werden [7, 9, 10, 18].

Alkoholkonsum scheint bei E‑Scooter-Unfällen eine weitere wichtige Rolle zu spielen. 22 (36,7 %) der in die vorlegte Studie eingeschlossenen Patienten waren zum Zeitpunkt des Unfalls alkoholisiert. Bei genauer Betrachtung der Tageszeit ereigneten sich 16 (72,7 %) dieser 22 Unfälle nachts zwischen 22 und 6 Uhr. Die in dieser Studie relativ hohe Quote von 36,7 % unter Alkoholeinfluss verunfallter E‑Scooter-Fahrer liegt im oberen Feld der vergleichbaren Studien, die eine Quote an alkoholisierten E‑Roller-Fahrern zwischen 5,2 und 48 % dokumentiert haben [9, 10, 12]. Dies lässt den Schluss zu, dass E‑Scooter gerade nachts bei jungen Menschen als leicht verfügbare und schnelle innerstädtische Transportalternative gesehen werden. Diese nächtliche Leichtsinnigkeit kann, wie unsere Ergebnisse zeigen, für den Einzelnen drastische gesundheitliche Konsequenzen nach sich ziehen.

Die in unserer Studie aufgetretenen Verletzungen wurden nach Körperregion aufgeschlüsselt; dies ergab eine Verteilung von 52 % (*n* = 53) auf den Kopf- bzw. Gesichtsbereich, 26,5 % (*n* = 27) auf die obere Extremität, 16,7 % (*n* = 17) auf die untere Extremität und 4,9 % (*n* = 5) auf den Körperstamm. Diese Verteilung deckt sich sehr stark mit bislang publizierten Daten [9, 10, 19]. Eine rezente biomechanische Analyse, in der Crash-Tests mit Dummys durchgeführt wurden, wird durch die Ergebnisse der vorliegenden Studie wiederum bestätigt. Die Kopfregion war auch hier die am häufigsten betroffene Körperregion, gefolgt von der oberen Extremität, die v. a. durch den Versuch den drohenden Sturz abzufangen betroffen war [20].

Die Daten der aktuellen Studie zu der Notwendigkeit einer operativen Versorgung (25 %, *n* = 15) liefern im Vergleich zur gängigen Literatur eine im Mittelfeld liegende Quote, hier werden Operationsnotwendigkeiten zwischen 7 und 21 % beschrieben [9, 12, 18, 19].

In 16,7 % (*n* = 10) der Fälle mussten die Patienten direkt nach dem Unfall hospitalisiert werden; dies fällt im Vergleich zur Studie von Trivedi et al. aus den USA, der eine stationäre Aufnahme in 5,2 % beschrieb, deutlich höher aus [9]. Diese Diskrepanz ist am ehesten auf die grundlegenden Unterschiede zwischen dem US-amerikanischen und dem deutschen Gesundheitssystem in Bezug auf eine stationäre Aufnahme zurückzuführen [21].

Mit einem mittleren ISS von 3,8 waren die in dieser Studie erlittenen Verletzungen durch E‑Scooter-Unfälle im Durchschnitt als leicht zu werten. Es muss jedoch betont werden, dass in der vorliegenden Studie 2 (3,3 %) schwer verletzte Patienten mit einem ISS über 15 registriert wurden. Dies korreliert mit den von Kobayashi et al. beschriebenen Daten (ISS >15 in 6 % der Fälle) [10].

Bei der Diskussion von Verletzungen bei E‑Scooter-Unfällen sind gerade bei einer Krankenhausverweildauer bis zu 26 Tagen (RW: 1–26, Median: 3,5) die entstehenden sozioökonomischen Kosten nicht zu vernachlässigen. Gerade die hier typischen Traumamechanismen, Stürze mit bis zu 20 km/h, in der Mehrzahl der Fälle ohne Tragen eines Helms bedürfen einer gründlichen Diagnostik in der Notaufnahme mit hohem technischen und personellen Aufwand, vergleichbar zu einer Schockraumbehandlung. Zudem ist eine OP-Bereitschaft auch nachts vorzuhalten und als weiterer Kostenfaktor für das Gesundheitswesen anzusehen [22]. Im Vergleich zu Daten zur Gesundheitsökonomie bei E‑Scooter-Unfällen muss davon ausgegangen werden, dass durch die vermutlich steigende Anzahl an Unfällen gleichzeitig auch die entstehenden Kosten ansteigen könnten[8, 10, 18].

Unsere Studienpopulation ergab nur einen Verletzten, der nicht selbst den E‑Scooter bediente. In der Literatur wird insgesamt von mehreren Fällen verletzter Fußgänger oder anderer Straßenteilnehmer berichtet. Dies ist besonders darauf zurückzuführen, dass E‑Scooter unerlaubterweise oft auf Gehwegen benutzt werden und die Geschwindigkeit des E‑Rollers unterschätzt und so andere Straßenteilnehmer gefährdet werden [23, 24].

Da das Oktoberfest als weltgrößtes Volksfest jährlich mehrere Millionen Menschen nach München lockt, die auch hier dem Alkohol nicht abgeneigt sind, möchten wir diese 2‑wöchige Zeitspanne noch gesondert hervorheben. Neun Patienten stellten sich nach dem Oktoberfestbesuch und E‑Scooter-Unfall in unserer Notaufnahme vor (Abb. 6), wobei zwei Drittel der Patienten alkoholisiert waren. Im Durchschnitt verletzten sich die Patienten zudem während des Oktoberfests (Mittelwert ISS: 6,7 vs. 3,3) schwerer im Vergleich zum restlichen Jahr. Zwei der Patienten waren zudem schwer verletzt (ISS von 16 und 24). Der Unfallzeitpunkt und der Alkoholisierungsgrad korrelierten gleichzeitig nicht miteinander. Ein Helm wurde von keinem Patienten getragen.

Diese Studie birgt einige Limitationen. Es kann nicht sicher ausgeschlossen werden, dass die Datenerhebung der Verunfallten E‑Scooter-Nutzer/-innen lückenhaft erfolgte. Zudem sollte im Hinterkopf behalten werden, dass diese Studie ab Beginn der Einführung in Deutschland, wo die Neugier auf das neue Transportmittel besonders groß war, während der Oktoberfestzeit und im Lockdown der Coronakrise durchgeführt wurde und so eine Varianz der Nutzung von E‑Scootern vorliegen kann.

## Fazit für die Praxis

- Die in unserer Studie erhobenen Daten lassen in Zusammenschau mit den vorhandenen Daten aus den USA in den kommenden Jahren eine weitere Zunahme an E‑Scooter-Unfällen antizipieren. Auch wenn die Anzahl der E‑Scooter-Unfälle (*n* = 60), verglichen mit den Fahrradunfällen (*n* = 622), die im gleichen Zeitraum in der Notaufnahme am Klinikum rechts der Isar vorstellig wurden, relativ gering erscheint, ist von einem erheblichen personellen als auch diagnostischen Aufwand bei der Versorgung von E‑Scooter-Verunfallten auszugehen. Eine gründliche und strukturierte klinisch-radiologischen Abklärung mit dem Fokus auf Kopf und HWS-Verletzungen sollte in Anbetracht der von uns ermittelten Daten als unabdingbar angesehen werden.
- Zudem sollte in Anbetracht der großen Anzahl an Kopfverletzungen eine generelle Helmpflicht erwogen werden, oder mindestens eine Option eines Helmverleihs in Verbindung mit der E‑Scooter-Miete ermöglicht werden. Technisch könnte dies wie bei Sharing-E-Fahrrädern über einen am E‑Scooter montierten Helmkoffer erfolgen.
- Hier sollten die politischen Verantwortlichen, Vermieter und Verkehrsclubs im Zuge der Lockerung der Ausgangsbeschränkungen intensivere Informationskampagnen starten und auf die Gefahren hinweisen. Des Weiteren könnten nächtliche Alkoholkontrollen bei E‑Scooter-Fahrern, wie im regulären motorisierten Straßenverkehr üblich, durchgeführt werden und so zur Unfallprävention beitragen.

## Abkürzungen

AC-Gelenk: Akromioclaviculargelenk
ADAC: Allgemeiner Deutscher Automobil-Club
BMVI: Bundesministerium für Verkehr und digitale Infrastruktur
DGOU: Deutsche Gesellschaft für Orthopädie und Unfallchirurgie
E-Scooter: Elektro-Scooter
ISS: Injury Severity Score
LWK: Lendenwirbelkörper
MW: Mittelwert
OMT: Os metatarsale
RW: Reichweite
